# Human primary epidermal organoids enable modeling of dermatophyte infections

**DOI:** 10.1038/s41419-020-03330-y

**Published:** 2021-01-04

**Authors:** Xuan Wang, Shuyong Wang, Baolin Guo, Yuxin Su, Zuolong Tan, Mingyang Chang, Jinmei Diao, Yi Zhao, Yunfang Wang

**Affiliations:** 1 Translational Medicine Research Center, Beijing Tsinghua Chang Gung Hospital, Beijing, 102218 China; 2 Department of Stem Cell and Regenerative Medicine, Beijing Institute of Health Service and Transfusion Medicine, Beijing, 100850 China; 3 Army Tuberculosis Prevention and Control Key Laboratory, Beijing Key Laboratory of New Techniques of Tuberculosis Diagnosis and Treatment, Institute for Tuberculosis Research, the 8th Medical Center of Chinese PLA General Hospital, Beijing, 100091 China; 4 Department of Dermatology, Beijing Tsinghua Chang Gung Hospital, School of Clinical Medicine, Tsinghua University, Beijing, 102218 China

**Keywords:** Skin stem cells, Infection

## Abstract

Technology of generating human epidermal derivatives with physiological relevance to in vivo epidermis is continuously investigated for improving their effects on modeling of human natural dermatological status in basic and clinical studies. Here, we report a method of robust establishment and expansion of human primary epidermal organoids (hPEOs) under a chemically defined condition. hPEOs reconstruct morphological, molecular, and functional features of human epidermis and can expand for 6 weeks. Remarkably, hPEOs are permissive for dermatophyte infections caused by *Trichophyton Rubrum* (*T. rubrum*). The *T. rubrum* infections on hPEOs reflect many aspects of known clinical pathological reactions and reveal that the repression on IL-1 signaling may contribute to chronic and recurrent infections with the slight inflammation caused by *T. rubrum* in human skin. Thus, our present study provides a new insight into the pathogenesis of *T. rubrum* infections and indicates that hPEOs are a potential ex vivo model for both basic studies of skin diseases and clinical studies of testing potential antifungal drugs.

## Introduction

The mammalian skin epidermis comprises a highly specialized and multi-layered epithelium that includes the stratum basale, stratum spinosum, stratum granulosum, and stratum corneum, which protects the body against harmful factors^1^. In the past few decades, classical two-dimensional (2D) culture methods^2–4^ have been used as invaluable tools to supply primary epidermal cells for studies on skin stem cell biology as well as for stem cell-based therapies of extensive and severe skin injuries^5–7^. However, these 2D culture techniques for human epidermal cells rely on the presence of feeder layers of murine fibroblasts (3T3 cells) and media supplemented with fetal bovine serum or bovine pituitary extract^2–4^. These conditions introduce unidentified variables or xenobiotic substances that can bring unfavorable impacts on clinical safety and can also interfere with the mechanistic studies on skin diseases. Hence, safer and more-effective culture systems according to clinical settings have been investigated for decades^8^. Until recently, the chemically defined and xeno-free culture methods have been successfully developed for long-term culture of human epidermal cells with the use of small molecules^9^ or laminin-based matrices^10^. However, these expanding epidermal cells in 2D format cannot recapitulate the in vivo cellular composition and the physiological structure of human epidermis. Over the past decades, several protocols have been developed to efficiently generate three-dimensional (3D) skin equivalent with physiological relevance to human skin^11^, especially the reconstructed multi-layered epidermis under the air–liquid interphase (ALI) condition^12–14^, which have provided elaborate skin 3D models for skin research and regenerative medicine. However, the reconstructed epidermis using ALI method have a limited lifespan and passage ability, which may limit large-scale application of this model system for industry or clinical programs.

Recently, the established technologies for 3D organoid cultures have enabled the robust generations of various mini-organs that resemble their tissue-of origin, and have also allowed for the long-term expansions of these mini-organs under the conditions of chemically defined systems^15,16^. These organoid-associated techniques show many great advantages for generating model systems of tissues including small intestine^17^, stomach^18^, brain^19^, liver^20^, kidney^21^, and esophagus^22,23^. Likewise, the organoid technology provides an opportunity for generating 3D model system of skin tissue. Notably, we and others have successfully established several different type of skin-associated organoids, such as mouse and human hair-bearing organoids^24,25^, canine and mouse epidermal organoids^26,27^, mouse sweat gland organoids^28^, and sebaceous gland organoids^29^. Until now, however, a method for the efficient establishment and effective culture of human epidermal organoids as a model of human epidermis tissue has not yet been reported. In addition, there is still no report of human epidermal organoids applied in the studies on skin diseases.

Here, we report the extensive and reproducible establishment of human primary epidermal organoids (hPEOs) from human interfollicular epidermis. hPEOs are expandable and contain both proliferative epidermal stem/progenitor cells and differentiated epidermal cells. They have a highly organized structure resembling human epidermis. Remarkably, hPEOs can be established from single Integrin α6^high^ cells through clonal expansion and cell differentiation. For proving their applications in analyses of skin infectious diseases, hPEOs were used to model *T. rubrum* infections that are the most prevalent dermatophyte to cause human nail and skin infections worldwide^30,31^. The *T. rubrum* infections on hPEOs reflected many aspects of known clinical pathological reactions. Furthermore, we found that *T. rubrum* triggered the upregulated expression of the anti-inflammatory factor, IL-1RN (IL-1 receptor antagonists) at transcriptional, translational, and secretion levels in the infected cells. These induced antiinflammation effects by *T. rubrum* may be responsible for its high degree of adaptation to human skin and for its tendency to cause chronic infections with slight inflammation. Thus, our study provides the first-hand evidences for application of hPEOs as a novel system to model skin infectious diseases and also provides a theoretical basis for antifungal treatments in future.

## Results

### Establishment of hPEOs as ex vivo human epidermal derivatives

**Fig. 1:**
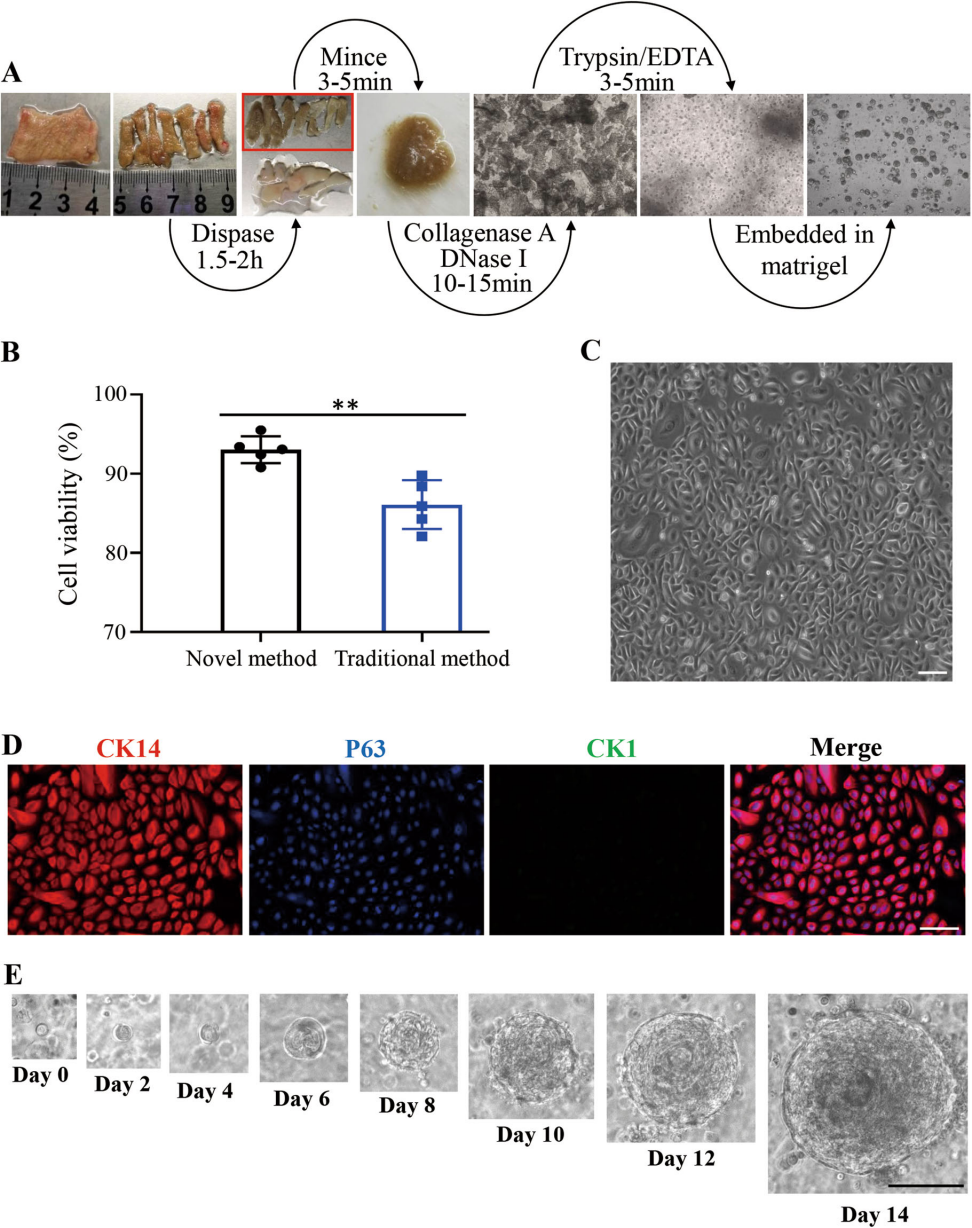
A novel human epidermal cells isolation system and growth of hPEOs. **A** Schematic representing isolation of epidermal cells from human foreskin tissue. **B** Comparison of the viability of cells derived from the traditional method and the novel method. Results are the mean ± SD from five independent repeated experiments. n.s., not significant (*p* > 0.05), *p < 0.05, **p < 0.01, ***p < 0.001. **C** Morphology of isolated human epidermal cells after attachment in a low-calcium, serum-free medium (EpiLife). **D** Immunofluorescence analysis of epidermal progenitor cell markers, CK14 and P63, in the attached epidermal cells. **E** Representative serial images of hPEOs growing at the indicated time points in the NaNBEFNoRWAFs medium. SD, standard deviation. Scale bar: 50 µm (**C**, **D**), 100 µm (**E**).

Previously, the commonly used protocol (or traditional method) to isolate epidermal cells includes an overnight procedure for tissue digestion, which is time-consuming and may led to decreased cell viability and lowered viable cell yields^32^. In addition, there also existed reports of fast methods to isolate keratinocytes^7,33^. However, all of these previously reported methods have specifically applied scenarios. For instance, the traditional method usually works well for neonatal tissues, but it becomes very difficult to use when used to isolate cells from adult tissues^32^. The reported fast method used in RECELL system is suitable for isolating epidermal cells from the thin split-thickness cutaneous biopsy (0.2–0.3 mm) harvested from an uninvolved area using a dermatome^33^. And other trypsin-ethylenediaminetetraacetic acid (EDTA)-based fast method usually produces a heterogeneous cell population, including keratinocytes and fibroblasts^7^. It is unclear whether these methods function well enough to efficiently isolate epidermal cells from human skin specimens with irregular thickness and size from various clinic scenario, such as the specimen from circumcision, abdominal operation or plastic surgery. We therefore started to develop a fast and robust protocol via an organized procedure with the sequential usages of various enzymes for specifically digesting skin tissue extracellular matrix protein in order to isolate pure human epidermal cells from foreskin tissues within 3 h (Fig. 1A). Based on our novel method with significant time saving, the cell viability was improved with statistical significance, when compared with the reported traditional method^32^ (Fig. 1B and Supplementary Fig. 1). In 2D culture at day 7, the isolated human epidermal cells displayed a typical cobblestone-like morphology under the condition of a commercially available medium (EpiLife medium) without support from feeder cells (Fig. 1C). Most of the attached cells were positive for basal stem/progenitor cell markers, CK14 and P63, and negative for suprabasal cell differentiation marker CK1 (Fig. 1D). Taken together, all of above results indicated that our newly established isolation protocol had the advantage of improved efficiency for isolation of viable epidermal cell populations containing basal stem/progenitor cells.

Next, we tried to use the freshly isolated human primary epidermal cells to establish 3D cultures of hPEOs. Because the inter-species differences exist between human skin and canine or mouse skin^34^, all of previously reported culture methods for mouse and canine epidermal organoids^26–28^ could not be satisfactorily used for human epidermal organoid culture. Therefore, a new culture method with required components for culturing hPEOs was totally established. Candidate components were identified from five primary categories consisting of 10 factors: *N*-acetylcysteine (NAC, here abbreviated as Na), N2 (N), B27 (B), EGF (E), FGF-10 (F), Noggin (No), R-Spondin1 (R), Wnt3a (W), A83-01 (A), and Forskolin (Fs), which were collectively referred to as NaNBEFNoRWAFs. The major reasons to select these factors were based on our specially designed principles: 1. antioxidants have been reported to protect epidermal stem cells from aging and to maintain their stemness phenotype^35^. *N*-acetylcysteine was chosen because of its antioxidant activity and its inhibitory effects on apoptosis of epithelial cells^36^ and on epithelial-mesenchymal transition^37^; 2. the serum-free supplements, both N2 and B27, were selected for their roles to support organoid cultures without addition of serum^38^; 3. the activation of morphogen signaling pathways such as epidermal growth factor receptor, keratinocyte growth factor receptor, and Wnt/β-catenin signaling have been demonstrated to promote epidermal morphogenesis and proliferation^39–41^. The relevant activators of these pathways like EGF, FGF-10, Wnt3a, and R-Spondin 1 were therefore selected; 4. dual SMAD signaling inhibition enabled expansion of epidermal stem cells in 2D culture^9^, therefore A83-01 (TGFβ inhibitor) and Noggin (BMP antagonist) were included. 5. the increased level of cellular cAMP (cyclic adenosine monophosphate) promoted the proliferation of epidermal cells^42^. Forskolin is capable of up-regulating intracellular cAMP by activating adenylyl cyclase and so was selected. Under the specially designed conditions with the ten factors, the freshly isolated human epidermal cells that were embedded in basement membrane extract (BME) or matrigel proliferated rapidly, and formed solid organoids ~200 µm in diameter with a concentric cell arrangement within ~14 days (Fig. 1E).

### Optimization of culture conditions for hPEOs

**Fig. 2:**
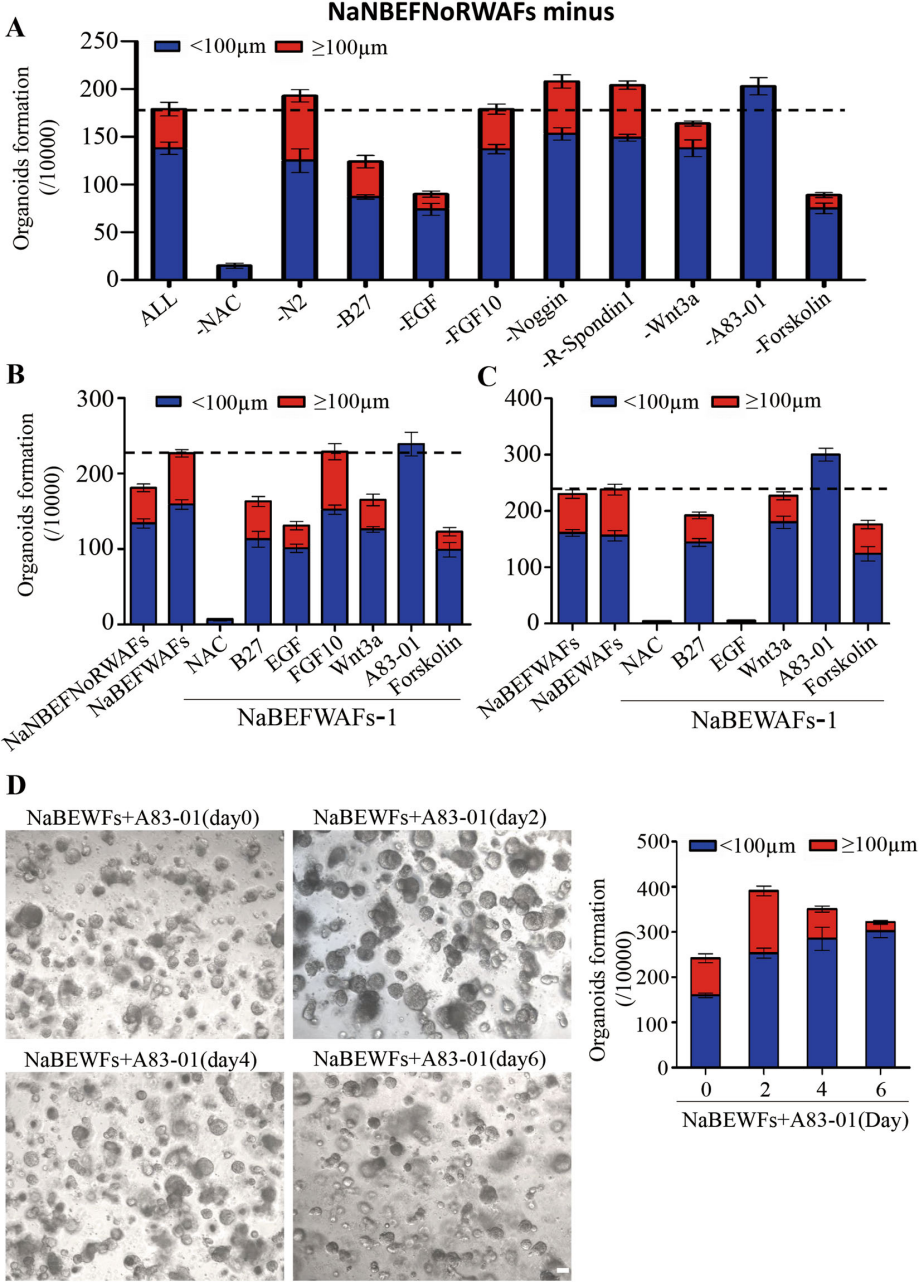
Optimization of hPEOs cultures. **A** The numbers of hPEOs after removal of individual factors from the NaNBEFNoRWAFs pool. **B**, **C** The numbers of hPEOs were counted after the removal of individual factors from the pool of NaBEFWAFs (**B**) and NaBEWAFs (**C**). **D** Representative images of hPEOs generated by various timings of A83-01 treatment in NaBEWFs medium. The hPEOs were counted on day 10. Results are the mean ± SD from three replicates from three independent repeated screening experiments. All screening experiments were performed with keratinocytes from three different donors. Scale bar: 100 µm (**D**).

The results from above experiments suggested the clues for optimizing the combination of small molecules or factors for generation and culture of hPEOs. Therefore, each of above-selected candidate was further analyzed individually and dynamically. During our studies, we removed factors one by one from the pool of NaNBEFNoRWAFs to test its effect individually. Removal of each of N2 (N), Noggin (No), or R-Spondin 1 (R) resulted in the increased number of total hPEOs and large-size hPEOs (≥100 µm in diameter). Removal of A83-01 also increased the total number but decreased the size for all of generated hPEOs (<100 µm in diameter). On the contrary, both the total number and the number of large-size hPEOs were decreased significantly after removing each of NAC (Na), B27 (B), EGF (E), Wnt3a (W), or Forskolin (Fs) (Fig. 2A and Supplementary Fig. 2).

Elimination of FGF-10 did not significantly alter the quantity of generated hPEOs. However, we still preserved it in selection group until the next round of removal test because FGF-10, as a member of FGF7 subfamily, has a key role in promoting the proliferation of basal epidermal cells^43,44^. Therefore, NAC, B27, EGF, FGF-10, Wnt3a, A83-01, and forskolin (NaBEFWAFs) were collected and further analyzed in subsequent culture studies. Under the condition of treatment with NaBEFWAFs, both the total number of hPEOs and the ratio of large-size hPEOs were increased, as compared to those hPEOs under the condition of treatment with NaNBEFNoRWAFs (Fig. 2B). Two additional rounds of removal test finally proved that the collected candidates of NAC, B27, EGF, Wnt3a, A83-01, and Forskolin were essential for hPEOs formation (Fig. 2B, C and Supplementary Fig. 3 and 4). Particularly, removal of A83-01 increased total number of hPEOs, but mainly generated the small-size hPEOs in all dropout experiments. Therefore, we hypothesized whether A83-01 could have an effect on the generation of hPEOs in a time-dependent manner. To determine this, A83-01 was added into the pool of NaBEWFs on Days 0, 2, 4, and 6, respectively. Significantly, when A83-01 was added on Day 2, the combined pool induced the maximum efficiency on increases of numbers for both total and large-size hPEOs, though still with some heterogeneity in size between organoids (Fig. 2D). We, therefore, kept on employing this optimized combination to generate hPEOs for the subsequent studies.

### hPEOs maintained normal expressions of epidermis-specific markers

**Fig. 3:**
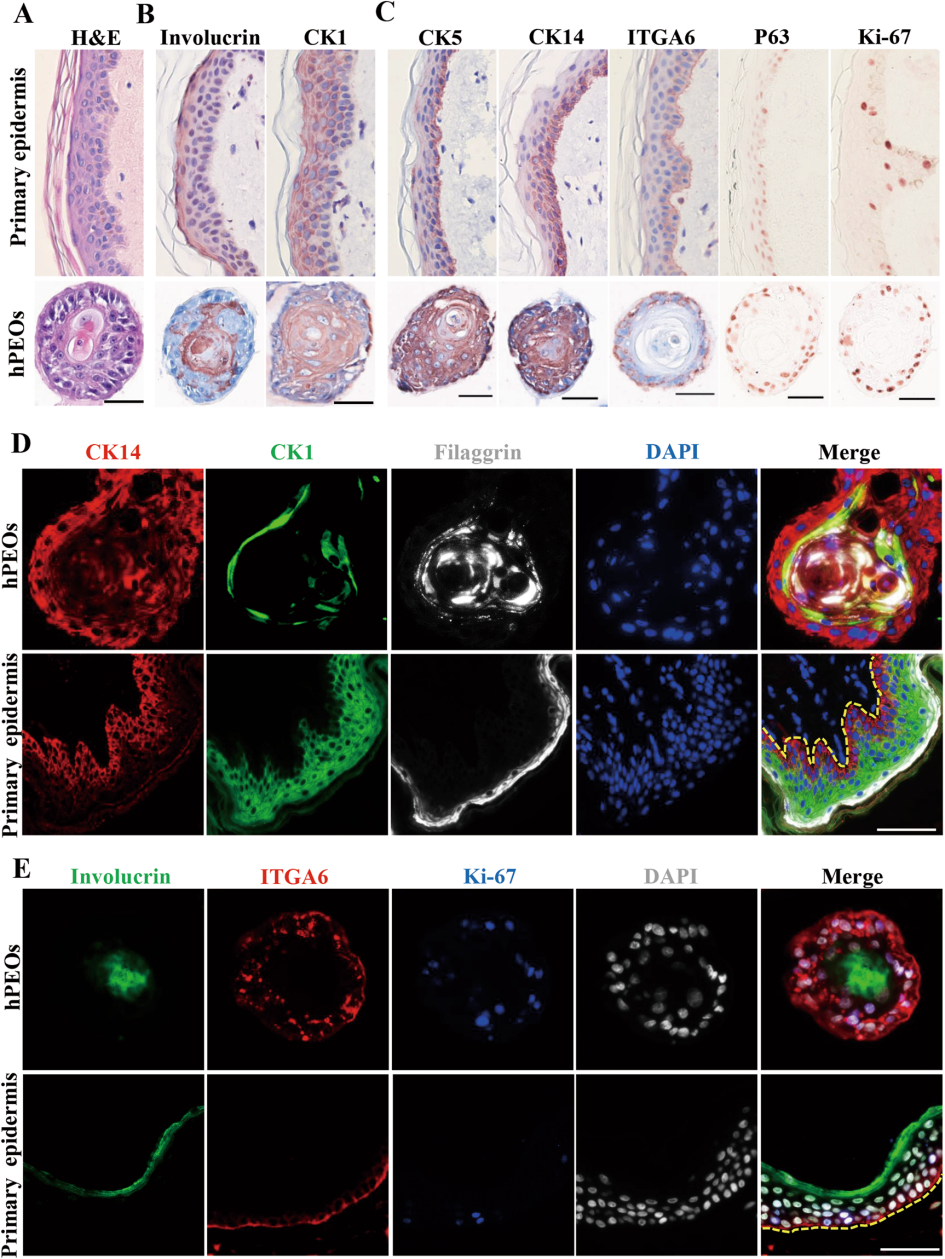
Marker expression of hPEOs. **A** H&E staining of the sections of hPEOs and human primary epidermis tissue. **B**, **C** Immunohistochemical staining for epidermal differentiation (**B**), stem cell-associated (**C**), markers of sections of hPEOs and human primary epidermis tissue. **D**, **E** Co-immunostaining analysis of various basal and differentiation markers in hPEOs and human primary epidermis tissue. Dotted line marks the epidermis–dermis junction. Scale bar: 50 µm.

Next, the histological characteristics of hPEOs were analyzed to compare with those of human primary skin tissue. Results of Hematoxylin and Eosin (H&E) staining indicated that hPEOs were morphologically similar to normal skin epidermis after 10 days in culture with small basal-like cells in contact with the extracellular matrix; large flat suprabasal-like cells in the interior; and hardened keratinized material in the center of organoid structures (Fig. 3A). Afterwards, the cellular composition of hPEOs was compared with that of human primary skin tissue by using both basal cell and suprabasal cell-specific markers. Results indicated that the interior of hPEOs consisted of the differentiated cells shown by CK1^+^ immunostaining, as well as the abundant keratinization evidenced by Involucrin^+^ immunostaining (Fig. 3B). The outermost cell layer of hPEOs was positive for all analyzed stem/progenitor cell markers (CK5, CK14, integrin α6 (ITGA6), P63, and Ki-67), which was similar to their expression in the basal cells found in human skin epidermis (Fig. 3C). Moreover, results of co-immunostainings of stem/progenitor cell markers and differentiation markers also showed that hPEOs consisted of the stratified epithelial cells with both correct cellular architecture and markers resembling human skin epidermis: CK14, ITGA6, Ki-67, Integrin β4 (ITGB4), CK5, and SOX9 were expressed mainly in the basal layer; CK1 and CK10 were located in the suprabasal layer; filaggrin and involucrin were found in the central layer (Fig. 3D, E and Supplementary Fig. 5A, B). In addition, the main components of basement membrane (laminin, type IV collagen, and type VII collagen) were found in the base of hPEOs (Supplementary Fig. 5C). As expected, in all hPEOs, there were no detectable expressions for melanocyte marker, Gp100, Langerhans cell marker, CD1a, vascular cell marker, CD31, and the marker for fibroblasts, Vimentin (Supplementary Fig. 5D). Together, these data demonstrated that hPEOs, as the human primary skin tissue-derived epidermal organoids, have a well-differentiated, keratinized stratified squamous epithelium.

### hPEOs primarily originated from basal cells

**Fig. 4:**
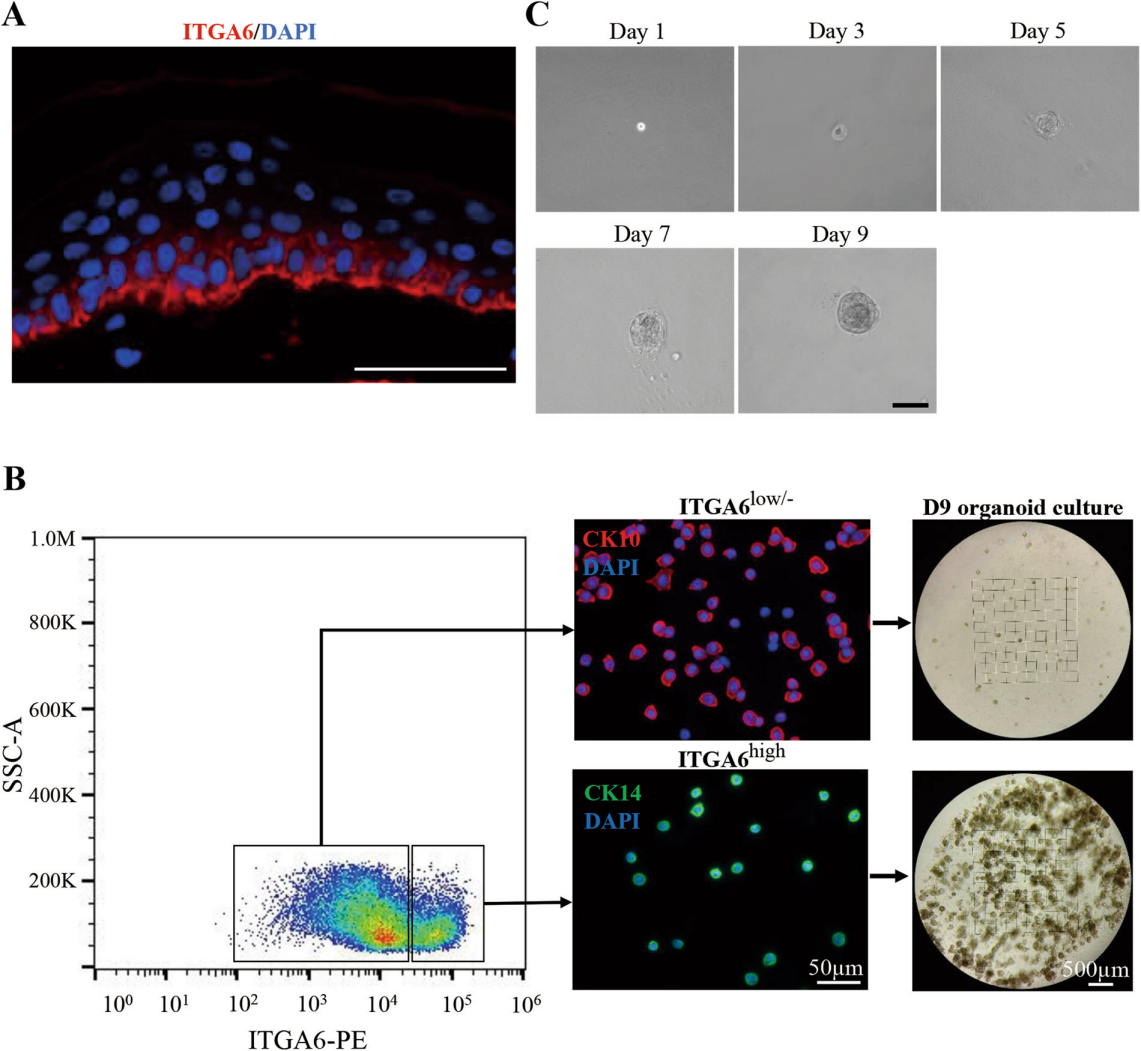
The hPEOs derived from basal layer cells. **A** ITGA6 immunostaining of human primary epidermis tissue counterstained with DAPI. **B** Human epidermal cell suspensions were separated into ITGA6^high^ cells and ITGA6^low/−^ cells. Identity of the populations was confirmed by staining for CK14 and CK10. Sorted cells were grown in organoid cultures for 9 days. **C** Representative serial images of an epidermal organoid generated from single ITGA6^high^ cells. Scale bar: 50 µm (**A**), 100 µm (**C**).

Furthermore, we pursued the study to investigate whether some special subpopulations of human epidermal cells could act as the cell origin for hPEOs. In fact, mammalian skin epidermis in vivo contain the heterogeneous populations of epidermal cells including basal progenitors, differentiated suprabasal cells and granular cells. In line with previous finding^45^, our result of in situ immunostaining showed that ITGA6, as a surface marker of stem/progenitor cells in human skin epidermis, was located primarily in their basal layer (Fig. 4A). Therefore, ITGA6 was further employed for differentially sorting the freshly isolated human primary epidermal cells. Results of cell sorting analyses indicated that the ITGA6^high^ cells were primarily the CK14^+^ cells with a small, round shape, displaying the properties of basal progenitors, whereas the ITGA6^low/−^ cells were mainly the CK10^+^ cells with differentiation characters of a big and flat morphology (Fig. 4B). After fluorescence-activated cell sorting (FACS)-based cell sorting, the isolated ITGA6^high^ cells or ITGA6 ^low/−^ cells were cultured under the same condition for hPEOs formation and maintenance. Results indicated that the ITGA6^high^ cells developed into hPEOs much more efficiently than the ITGA6^low/−^ cells (Fig. 4B). Remarkably, after FACS isolation, a single ITGA6^high^ cell was fully competent to develop into a hPEO within 9 days (Fig. 4C). Together, these results strongly supported that hPEOs could originate primarily from the basal cells of human epidermis.

### The hPEOs showed moderate proliferative potential and maintained genetic stability during passaging

**Fig. 5:**
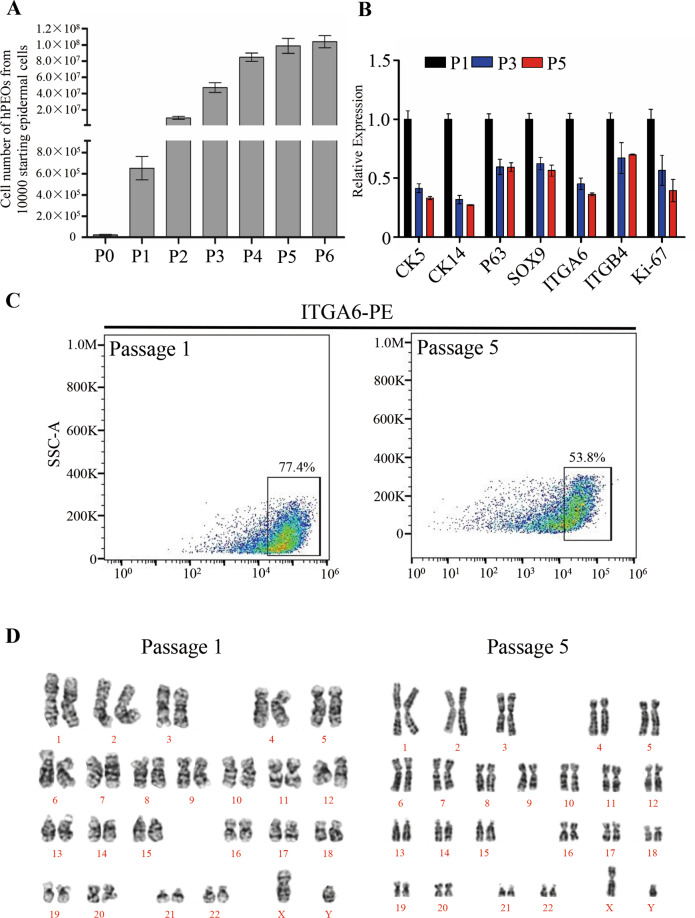
Expansion potential of hPEOs. **A** Cell numbers of hPEOs at each passage derived from ten thousand epidermal cells. Results are mean ± SD from three replicates from three independent repeated experiments. **B** qRT–PCR of the indicated markers in hPEOs of passage 1 and 5. **C** Representative FACS plots of ITGA6^high^ cells at passage 1 and 5. **D** Karyotype of hPEOs at passages 1 and 5 is shown.

Next, additional characteristics of hPEOs were further determined during long-term cultures. Remarkably, our hPEOs were found to expand for up to six passages, and they reached to a total amplification of 10,000-fold within 6 weeks (Fig. 5A). However, over sequential passaging, results of serial qRT–PCR analyses showed that the expression levels of basal cell markers (*CK5*, *CK14*, *P63*, *SOX9*, *ITGA6*, and *ITGB4*) decreased, and the level of proliferating cell marker Ki-67 was also downregulated (Fig. 5B). Reduction of the proportion of ITGA6^high^ cells was also observed during continuous passaging (Fig. 5C). Therefore, these results explained that the proliferation of hPEOs under present established conditions remained a possible limitation. During the five continuous passages ex vivo, the chromosomal stability was also evaluated by performing karyotype analysis, which showed that hPEOs maintained a normal karyotype continuously (Fig. 5D). Together, these results suggested that the present culture conditions for hPEOs were reliable and could be optimized for additional expansion after further improvements.

### Reconstruction of pluri-stratified epidermis equivalents from hPEOs

**Fig. 6:**
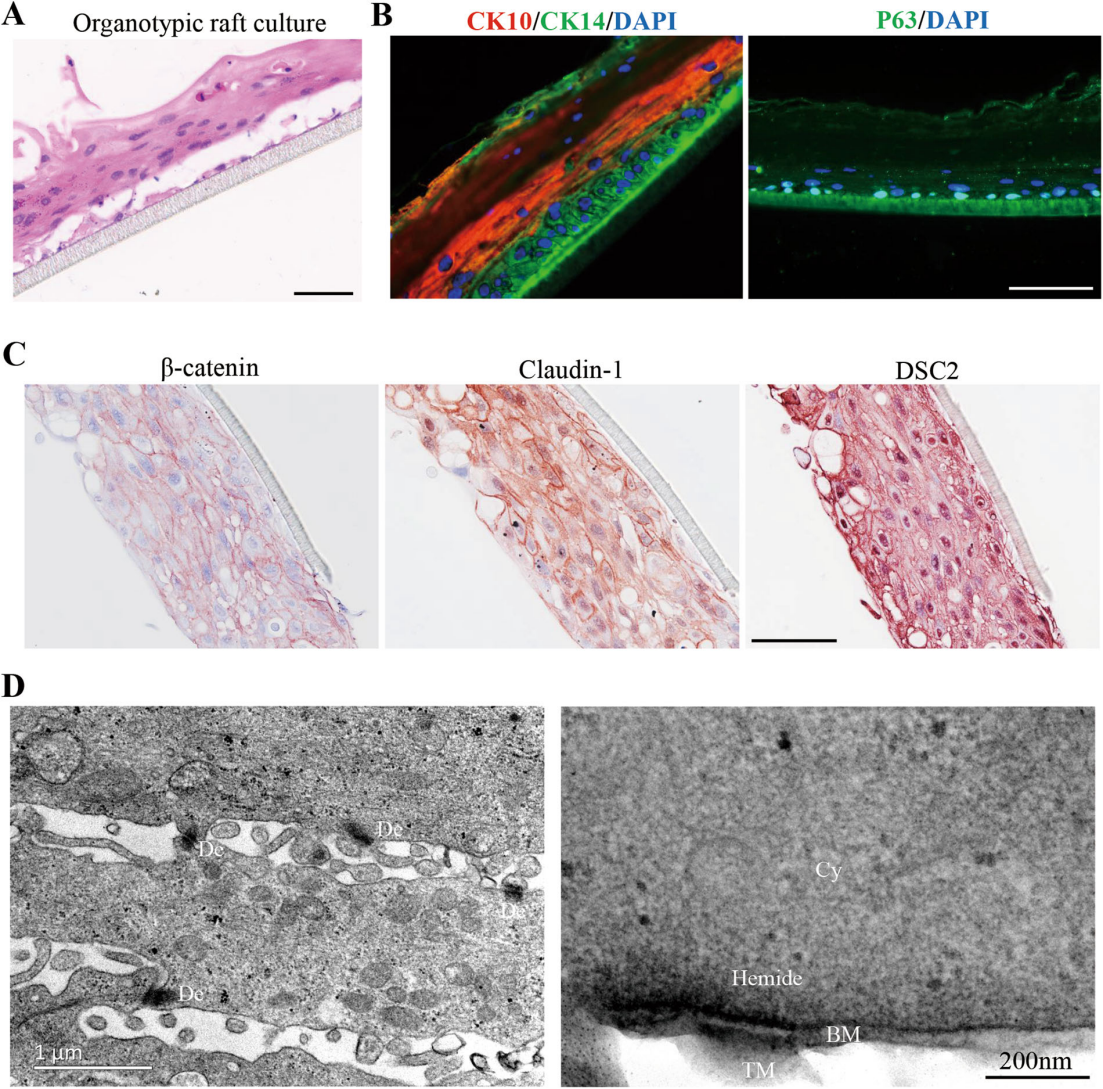
Development of a full-thickness human epidermis equivalent from hPEOs. **A** H&E staining of organotypic rafts generated from hPEOs. **B** Immunofluorescence analysis of the expression and localization of CK10, CK14, and P63 in hPEOs-derived organotypic rafts. **C** Immunohistochemistry of cell–cell junction markers on hPEO-derived organotypic rafts. DSC2, desmocollin 2. **D** Section TEM of organotypic rafts derived from hPEOs. *De* desmosomes, *Hemide* hemidesmosomes, *BM* basement membrane, *Cy* cytoplasm, *TM* transwell membrane. Scale bar: 50 μm (**A**–**C**).

Mammalian skin epidermis contains the basal stem/progenitors that can give rise to all of the differentiated stratified layers during homeostasis^46^. Here, we tested whether the hPEOs containing basal cells could have the capability to generate reconstructed human epidermis in vitro. Previously, the epidermis equivalents as organotypic rafts were all established from cultured epidermal basal cells in vitro under the ALI conditions^47^. Here, our hPEOs were enzymatically dissociated into single cells and exposed to ALI differentiation for 10–14 days. Results of H&E staining showed the generation of a pluri-stratified epithelium resembling primary epidermis (Fig. 6A). Results of immunostaining further showed that differentiation markers were normally expressed and located in layers. CK14^+^, P63^+^ cells were seen in the basal layer, whereas CK10^+^ cells were seen in the suprabasal layers (Fig. 6B). Moreover, an integrated network of cellular junctions is essential to support a stable environmental barrier and maintains the normal polarization of epidermis^48^. Notably, our results from immunostaining and ultrastructural analysis demonstrated that hPEO-derived epidermis equivalent possessed the correct distribution of adhesion junctions (β-catenin), tight junction (Claudin-1), desmosomes (DSC2), and hemidesmosomes (Fig. 6C, D). Together, these results suggested that hPEOs have the capacity to generate the stratified epithelium with the appropriate cellular architecture and to express markers resembling primary epidermis under ALI conditions.

### The hPEOs enabled for modeling dermatophyte infections

**Fig. 7:**
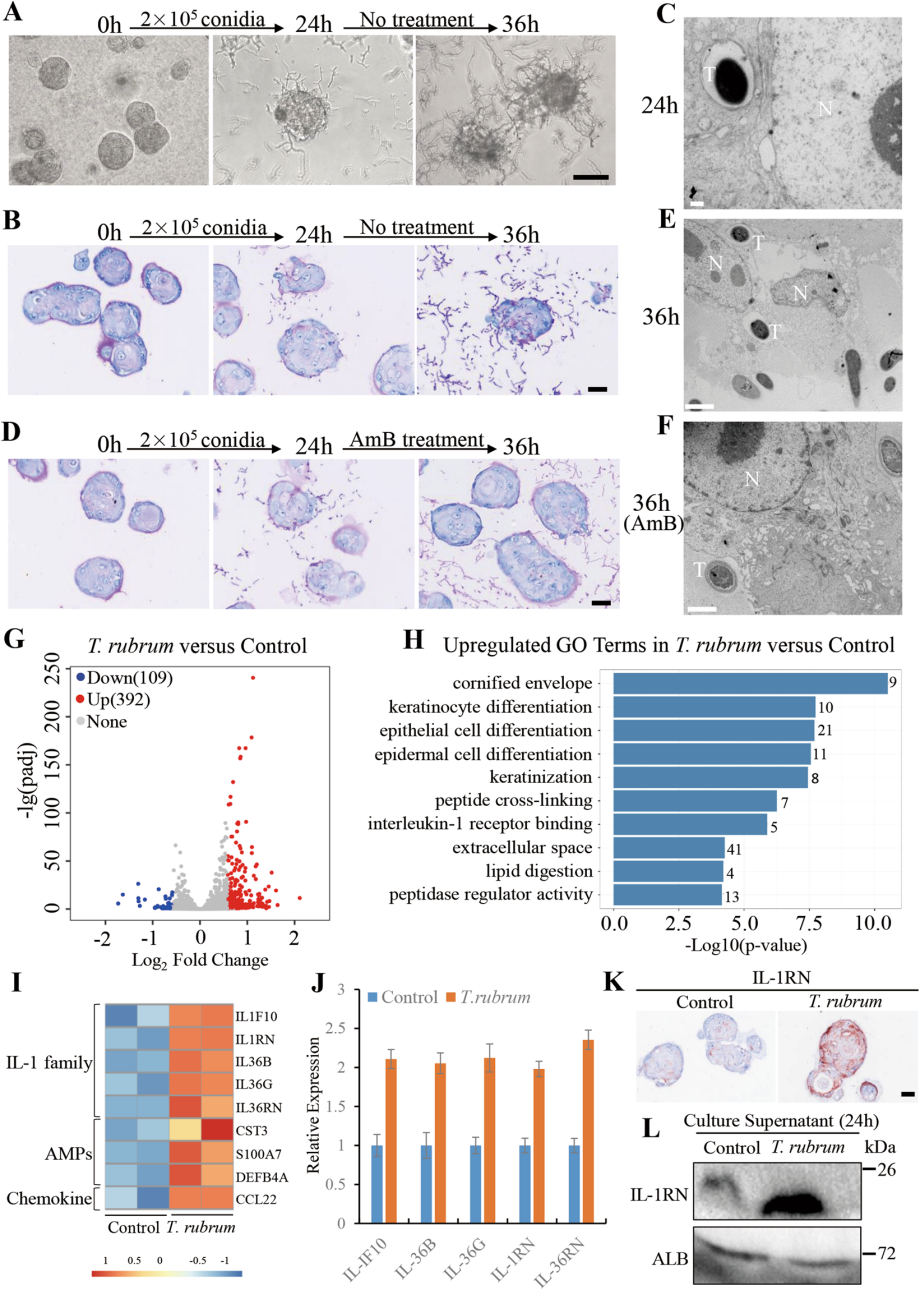
Modeling dermatophytosis in hPEOs. **A** Phase-contrast images showing progression of fungal elements on hPEOs infected by *T. rubrum* conidia over time. **B** Periodic-Acid Schiff (PAS) staining of infected hPEOs without antibiotic treatment. **C** The TEM of infected hPEOs after 24 h. **D** PAS staining of infected hPEOs with antibiotic treatment from 24 h. **E**, **F** The TEM of infected (**E**), antibiotic-treated (**F**) hPEOs after 36 h. **G** Volcano plot showing differential expression of host genes between *T. rubrum-*infected and control hPEOs at 24 h. Each dot represents a gene. The red and blue dots represent differentially expressed genes with *P* < 0.05. **H** GO analyses of upregulated genes in *T. rubrum* group versus control group. **I** Heatmap showing the expression of induced innate immunity-related genes. **J** qRT–PCR analysis of the expression of genes belonging to IL-1 receptor binding in *T. rubrum* group and the controls. **K** Immunohistochemical staining for IL-1RN in hPEOs of *T. rubrum* group and the controls. **L** Secretion lever of IL-1RN in culture supernatant of *T. rubrum* group and the control group at 24 h. AmB: amphotericin B. Scale bar: 100 μm (**A**), 50 μm (**B**, **D**, and **K**), 0.5 μm (**C**), 2 μm (**E**, **F**).

To further evaluate the potential application of hPEOs in studies of clinical dermatology, their capacities of host responses were tested after infections of *T. rubrum*. For inducing the infections, conidia of *T. rubrum* were used to inoculate hPEOs prepared in Matrigel. The conidia germinated gradually after inoculation, and a fraction of hyphal growth was observed successfully. Remarkably, some of hyphae adhered to hPEOs at 24 h and the extensive hyphae covered hPEOs at 36 h’ later (Fig. 7A). At the time of 24 h, results of PAS staining on the sections of infected hPEOs showed that some of hyphae invaded from the basal cells of hPEOs and penetrated into the hPEOs, whereas the organoid structure of hPEOs still remained relatively intact. At the time of 36 h post infection, the growing hyphae caused serious destructions of the structure of hPEOs (Fig. 7B). From results of transmission electron microscopy (TEM) imaging, the penetration of small numbers of *T. rubrum* hyphae into hPEOs was observed at 24 h, and the significant cell disruption was found at 36 h (Fig. 7C, E). In addition, inhibitory effects shown by the limited extent of fungal invasion were observed after 12-hour treatment with amphotericin B (AmB), a well-known antifungal agent (Fig. 7D, F).

We next investigated important genes involved in the host responses to *T. rubrum* using high-throughput RNA sequencing (RNA-Seq) of the infected organoids and control organoids. Given the situations of hPEOs integrity, and avoidance as much as possible for the contamination of the genome of *T. rubrum*, a period of 24 h of infection was considered appropriate for the evaluation by RNA-Seq. Therefore, transcriptomes of hPEOs at times of 24 h post infection, and at those on 24 h without infection were analyzed. Upon the infections of hPEOs, 392 genes were upregulated and 109 genes were downregulated (Fig. 7G). Gene ontology-term analysis of the upregulated genes revealed that a substantial number of genes were related to the cornified envelope, keratinocyte differentiation, keratinization, peptide cross-linking and lipid digestion, which contribute to the formation of the epidermal barrier (Fig. 7H). Representative upregulated genes belonging to the formation of a highly cross-linked, cornified envelope included involucrin (*IVL*), transglutaminases (*TGM1*, *TGM3*), corneodesmosin (*CDSN*), cornifelin (*CNFN*), keratins (*KRT33A*, *KRT34*, *KRT4*, *KRT7*, *KRT77*, *KRT78*, *KRT80*, *KRT84*), late cornified envelope proteins (*LCE1A*, *LCE1D*, *LCE1F*, *LCE2A*, *LCE2B*, *LCE2C*, *LCE2D*, *LCE3A*, *LCE3C*, *LCE3D*, *LCE3E*, *LCE6A*), small proline-rich protein (*SPRR2A*, *SPRR2B*, *SPRR2C*, *SPRR2D*, *SPRR2E*, *SPRR2F*, *SPRR2G*, *SPRR3*, *SPRR4*, *PRR9*), kallikrein related-peptidases (*KLK12*, *KLK13*, *KLK14*, *KLK6*, *KLK9*), serine protease inhibitors (*SERPINB12*, *SERPINB4*, *SERPINB7*), and desmoglein-4 (*DSG4*) (Supplementary Fig. 6A). These data suggested that *T. rubrum* triggered the stem/progenitor cells in hPEOs to undergo terminal differentiation and resulted in the formation of more mature stratum corneum, tight junctions and barrier lipids^49,50^, which are essential for the skin to minimize the effects of the infections. These phenomena are in accordance with clinical signs of thickened stratum corneum and desquamation observed in patients with *T. rubrum* infections.

In a parallel analysis, it was also known that infections of hPEOs also induced the increased expression of innate immune effectors in epidermal cells, which included upregulated antimicrobial peptides (AMPs)^51^ (*CST3*, *DEFB4A*, *S100A7*) and inflammation cytokines (*CCL22*, *IL1F10*, *IL-1RN*, *IL36B*, *IL36G*, *IL-36RN*) (Fig. 7I). Interestingly, many of those increased genes are associated with interleukin-1 (IL-1) receptor binding (Fig. 7H,I). These genes belonging to the IL-1 family^52^ further proved to be upregulated in hPEOs after *T. rubrum* infections by qRT–PCR (Fig. 7J). Active IL-1 signaling has been considered as a protective factor in skin responses to fungal infections^53^. As expected, our results showed the upregulation of pro-inflammatory cytokines of IL-1 signaling (e.g., *IL1F10*, *IL36B*, *IL36G*). Unexpectedly, we also found the increased expression of several anti-inflammatory cytokines, IL-1 receptor antagonists (*IL-36RN* and *IL-1RN*) (Fig. 7I, J). To reinforce this finding, protein expression of IL-1 receptor antagonists, IL-36RN, and IL-1RN, were tested further.

When compared with controls, results of both immunohistochemical analyses of organoid sections and western blot analyses of cell lysates of the infected hPEOs revealed that IL-1RN was significantly induced after *T. rubrum* infections, whereas IL-36RN did not change significantly (Fig. 7K and Supplementary Fig. 6B, C). Remarkably, *T. rubrum* also caused increased secretion of IL-1RN in the culture supernatant (Fig. 7L), suggesting that IL-1 signaling can be competitively inhibited at the receptor level by an antagonist(s) in *T. rubrum* infections. Our data suggested that *T. rubrum* may suppress the skin inflammation by releasing antagonists to compromise the pro-inflammatory IL-1 signaling, preventing inflammatory cascade reactions and evading the immune responses and elimination by human hosts. Generally, dermatophytes recognition is considered to be carried out by pathogen-associated molecular patterns in epidermal cells, especially cell surface toll-like receptors (TLRs) including TLR1, TLR2, TLR4, TLR5, TLR6, and TLR10^54,55^. We thereby started to investigate whether these receptors were potentially involved in mediating the interaction between *T. rubrum* and our organoids through qRT–PCR analysis of their expression before and after infection. As a result, the expression of TLR2 and TLR10 experienced no obvious alterations during infection, and the expression of TLR5 and TLR6 increased slightly, whereas the expression of TLR4 was downregulated significantly (Supplementary Fig. 6D), which were consistent with previous reports^56,57^. By contrast, the expression of TLR1 increased significantly after infection, indicating that it may be involved in initiating the cellular immune responses of organoids to *T. rubrum* infections. In addition, we also tested the expression of human beta defensins (hBD) including hBD1–4 in the organoids, which played important role in epidermal defense against dermatophyte. The results showed that the expression of all hBDs tested were upregulated after *T. rubrum* infections, especially the hBD-2 with the most significant upregulation (Supplementary Fig. 6E), suggesting that they also participated in epidermal defense against to *T. rubrum*.

In conclusion, our established infection model can well recapitulate the in vivo epidermal responses including epidermal differentiation and innate immune responses to *T. rubrum* infections and also revealed that the IL-RN produced by epidermal cells would be a contributing factor to the development of chronic and low inflammation in the infections caused by *T. rubrum*.

## Discussion

In this study, we developed a new method to successfully expand human epidermis as organoids for several passages, which provided a novel skin 3D model for skin research. Remarkably, hPEOs can be generated directly from freshly isolated keratinocytes within 10 days using our culture strategy, whereas classic reconstructed epidermis using ALI method need more time to be established because the freshly isolated epidermal cells need to expand to obtain large cell numbers for seeding on substrates before the start of ALI differentiation^12,13,58^. Therefore, hPEOs can be more efficiently produced than reconstructed epidermis for large-scale and rapid applications in industry or clinical programs. Furthermore, hPEOs can be generated even from the single, isolated ITGA6^high^ basal cells, and allow the epidermal proliferation and stratification at the same time, which cannot be achieved under ALI conditions. The clonogenic expansion of hPEOs from single cells can therefore provide an ideal model to study the epidermal stem cell homeostasis and mechanism of stratification at the single cells level. Nevertheless, we recognize that the reconstructed epidermis at ALI share more resemblance to the architecture of in vivo epidermis than our hPEOs.

Based on a newly established and chemically defined culture system without using bovine serum, murine feeder cells, and the bovine pituitary extract that are essential for classical 2D cultures^2–4^, our strategy and conditions for generating and expanding hPEOs are more conducive to those needed for clinical applications. Under our designed conditions, it was shown that a large quantity of human epidermal cells can be generated with a 10,000-fold expansion in 6 weeks. Generally, from a 1-cm^2^ biopsy, containing ~1−3 × 10^6^ cells, one can obtain at least 10^10^ human epidermal cells in <6 weeks and which maintain a normal karyotype in expanded cells. Such levels of cell numbers are sufficient for therapeutic applications for various human skin defects, particularly the large burn wounds. Moreover, the hPEOs generated in our culture system showed the moderate proliferation potential for up to six passages, which is similar to the level for expansion capacity of human epidermal cells cultured on classical feeders and in serum-containing medium^2^.

Previously, human primary epidermal cells or cell lines cultured in 2D state were used to model dermatophytosis and revealed that some pro-inflammation cytokines, antimicrobial peptides and the p-38 MAPK signaling were induced after dermatophyte infections^59–61^. In addition, the reconstructed human epidermis derivatives using ALI culture method were also employed for modeling dermatophytosis and testing antifungal treatments^57,62^. However, the mechanism underlying the *T. rubrum* infections with slight inflammation and tendency to become chronic; the adaption of *T. rubrum* to human immunological responses is still not fully understood^31^. Based on our established infection model, we obtained additional information about the mechanism that *T. rubrum* upregulated, such as the expression of an anti-inflammatory cytokine, IL-1RN, which may contributed to slight inflammation caused by *T. rubrum*, and to their then adaption to human hosts. In addition, previous studies showed that absent or dysfunctional responses by IL-1 receptor antagonists, e.g., IL-1RN, IL-36RN could resulted in the unregulated secretion of pro-inflammatory cytokines and caused auto-inflammatory skin diseases^63–65^, which also indicated the important role played by IL-1 receptor antagonists in controlling the intensity of skin inflammation. More importantly, a prior study uncovered that IL-1RN could also be upregulated in macrophages exposure to *T. rubrum*^66^, which was consistent with our finding as well. Based on our novel hPEOs model permissive for *T. rubrum* infections, we have provided a new explanation for long-standing questions in dermatophyte pathogenesis of chronic infection and also suggest that antifungal agents in combination with IL-1 agonists could contribute to complete the clearance of *T. rubrum* infections in patients.

Overall, hPEOs as a model system allow analysis of the epidermal cellular responses to dermatophyte infections directly and, in parallel, enable a display of the keratinization process at the same time. However, when co-cultured with organoids, dermatophyte have direct access to “basal” keratinocytes outside, and avoid the step of invasion into the stratum corneum, which prompt the process of infection to go directly into the stage of cellular responses and therefore cannot document the damage to epidermal barrier caused by *T. rubrum* infections. We must admit that the infectious process is not as physiological as in vivo situation and this is a limitation to our model as compared with ALI method-derived epidermis model infected with *T. rubrum*^67^. Indeed, our organoids are equipped with epidermal architecture and epidermal cellular microenvironment that can still faithfully reflect the in vivo epidermal responses triggered by *T. rubrum*. Nevertheless, the absence of immune cells in our organoids results in the inability of the model to recapitulate the complicated immunological responses to the infection exactly as it occurs in vivo, and this is a common defect that also exists in other previously described in vitro skin models, including infection on ALI method-derived epidermis^67^. Additional attempts on introducing the immune cells, such as Langerhans cells, neutrophils, macrophage and different type of lymphocyte, into the cultured organoids to construct immune-competent epidermal models will be helpful to investigate the complex cross-talk between epidermal cells and immune cells, as well as to better understanding of the immune responses of epidermal tissue to dermatophyte infections in the future.

In conclusion, our present study opens new approach for the applications of functional, human, primary epidermal organoids as the novel platforms for ex vivo studies, disease modeling, cell-based therapies and tissue engineering for the skin. The hPEOs are expected to serve as replacements or as supplemental model systems to the presently used ones required for feeder cell-based epidermal cells cultures, to more practically undertake the future skin biological and clinical studies.

## Methods and materials

### Human foreskin tissues

Human foreskin tissues were obtained from normal donors ranging in age from 12 to 60, following circumcisions performed at the 5th Medical center of Chinese PLA General Hospital with patients’ informed consent. Experiments were approved by the academic committee of the Beijing Institute of Health Service and Transfusion Medicine, and the ethics committee of the 5th Medical center of Chinese PLA General Hospital.

### Isolation of human primary epidermal cells from foreskin tissues

The subcutaneous fatty and connective tissues of human foreskin were removed as much as possible. Then, the tissues were cut into smaller pieces of 1 × 3 cm width and were digested with 2.5 U/mL Dispase (Gibco) for 1.5–2 h. After the rotating digestion, the epidermis was carefully peeled off by tweezers and cut into small pieces. The minced tissue pieces were subsequently digested with 2.5 mg/mL Collagenase A (Roche), 0.1 mg/mL DNase I (Sigma-Aldrich) for 10–15 min, and were manually shaken every 3–5 min. After centrifugation at 1000 rpm for 5 min, the pellets were subjected to 0.25% Trypsin-EDTA (Gibco) for a further 3–5 min. All of these digestions were maintained at 37 °C. After filtering through a 70 μm Nylon cell strainer (BIOLGIX) and centrifugation, we finally obtained human single, primary epidermal cells. The isolation of primary epidermal cells from foreskin tissue using traditional method was done according to protocols in a previous report^32^.

### Generation and expansion of hPEOs

The isolated epidermal cells were seeded in 30–50 μL BME 2 (R&D) or Matrigel (Corning) at a density of 1 × 10^2^−1 × 10^3^ cells/μL on ultra-low attachment surfaces of 24-well plates (Corning). After incubating the plates at 37 °C for 5–10 min for gelation, the droplets were cultured in Advanced Dulbecco’s Modified Eagle Medium/F12 medium supplemented with 0.1% bovine serum albumin (BSA), 1% N2, 1% B27, 10 mM HEPES, 1% GlutaMAX, 100 U/mL Penicillin, 0.1 mg/mL Streptomycin (all from Gibco), 1 mM *N*-Acetyl-l-cysteine (Sigma-Aldrich), 1 μM A83-01 (StemCell), 10 μM Forskolin (Selleck), 50 ng/mL EGF, 100 ng/mL FGF-10, 100 ng/mL Noggin, 250ng/mL R-Spondin 1, 100 ng/mL Wnt3a (all from R&D).

After testing each component in the culture medium, hPEOs were found to grow best in the optimized medium which was supplemented with 0.1% BSA, 1% B27, 10 mM HEPES, 1% GlutaMAX, 1 mM *N*-Acetyl-l-cysteine, 100 U/mL Penicillin, 0.1 mg/mL Streptomycin, 10 μM Forskolin, 50 ng/mL EGF, 100 ng/mL Wnt3a, and the 1 μM A83-01 that was added after 2 day’s culture. hPEOs could be removed from BME 2 or Matrigel by incubating the 3D drops on ice for 30–60 min and further dissociated into small clumps of cells or single cells with 0.25% Trypsin-EDTA. hPEOs were passaged at a 1:3–1:4 ratio every 5–7 days. hPEOs could be dissociated into small clumps or single cells and cryopreserved in serum-free cryopreservation medium (StemCell) and placed in −80 °C or liquid nitrogen, and also could be recovered with the optimized organoid medium.

### Isolation of ITGA6^high^ cells from primary human foreskin and organoid culture

Human epidermal cells were isolated as described above, and the cell suspensions were incubated with PE Rat Anti-Human ITGA6 (BD Biosciences) at 4 °C for 30–45 min. The stained cells were analyzed or sorted with BD FACSCalibur (BD Biosciences). The sorted cells were re-suspended in BME 2 and cultured with optimized organoid medium as described above. For the generation of hPEOs from a single ITGA6^high^ cell, the sorted ITGA6^high^ cells were embedded in Matrigel and seeded in 96-well plates at a density of 1 cell/well. Cells were cultured and expanded in the organoid formation medium as described above.

### Cell viability assays

BD Horizon™ Fixable Viability Stain 510 (FVS510) was used to evaluate cell viability according to the manufacturer’s instructions. The isolated keratinocytes obtained from different digestion methods were incubated with staining agent for 15–20 min at room temperature, protected from light, then washed three times with phosphate-buffered saline (PBS). The stained cells can be fixed with 4% PFA and sorted with a BD Horizon TM V500. The data were analyzed using the Flowjo software (TreeStar).

### Immunostaining

The 4% PFA-fixed skin tissues and organoids were embedded in paraffin, and 4 μm sections were prepared for immunostaining or H&E using standard protocols. For immunohistochemistry, slides were permeabilized with 0.25% Triton X-100 and blocked with 0.3% H_2_O_2_ solution, incubated with primary antibodies overnight at 4 °C, following with incubation with a secondary antibody for 1 h at room temperature. Subsequently, ABC and NovaRED staining were performed according to the manufacturer’s instruction (both from Vector Labs). For immunofluorescent staining, slides were permeabilized with 0.25% Triton X-100 and blocked with 10% Goat or Donkey serum in PBS for 1 h at room temperature. Then the slides were incubated with a primary antibody at 4 °C overnight. Secondary antibodies were incubated 1 h in the dark at room temperature. After counterstaining with 4′,6-diamidino-2-phenylindole (Sigma-Aldrich), the images were captured using Vectra 3.0.5 and processed using the Inform 2.2.0 (PerkinElmer). A complete list of the primary and secondary antibodies used is provided in Supplementary Table 1.

### Quantitative real-time PCR

Total RNA was isolated using RNeasy Micro or Mini Extraction Kit (Qiagen). Then 1 µg RNA was reverse-transcribed into cDNA using ReverTra Ace qPCR RT master mix (Toyobo, Japan) according to the manufacturer’s instructions. Quantitative real-time PCR (qRT–PCR) was performed on a Bio-Rad iQ5 System using the SYBR Green PCR Master Mix (Toyobo, Japan). Expression levels were normalized to the GAPDH. A complete list of the primers used is provided in Supplementary Table 2.

### Karyotype analysis

The hPEOs at passage 1 and passage 5 were incubated with 40 ng/mL colchicine (Sigma-Aldrich) for 4 h. Then the cells were dissociated into single cells with 0.25% Trypsin-EDTA and processed using a standard karyotyping protocol.

### Generation of reconstructed human epidermis from cells of hPEOs using ALI conditions

The hPEOs were dissociated into single cells, re-suspended in organoid medium, and subsequently plated onto 12-well Millicell Hanging Cell Culture Inserts (0.4 μm PET, Millipore) pre-coated with collagen I (rat tail, Corning) (5 µg/cm^2^) or Matrigel at a cell density of 5–8 × 10^5^/cm^2^. After 5 days of incubation, the cells were exposed to ALI by aspirating the medium from inside the culture inserts and then adding 1.2 mL of fresh medium consisting of complete EpiLife medium (Gibco) supplemented with 1.5 mM calcium chloride and 50 µg/mL vitamin C (Sigma-Aldrich) to the outside of each culture insert. The medium was refreshed every 2 days by removing the medium from the lower compartment and adding the fresh medium. The ALI cultures were maintained for 7–10 days.

### *Trichophyton rubrum* and production of conidia

Typical strain of *T. rubrum* were isolated from naturally infected skin of human who was previously diagnosed as tinea pedis and has been given written consent. The *T. rubrum* were grown on sabouraud dextrose agar at 25 °C for 2 weeks to reach confluence. Then the fungi were scraped and seeded over 3% oats/1.5% agar at 25 °C for 3 weeks. The surface of the plates was scraped and the scraping content was added into sterile PBS. The suspension was then filtered through 40 μm Nylon cell strainer in order to collect unicellular fungal elements corresponding to conidia. Then the conidia were centrifuged at 3000 rpm for 20 min at 4 °C, washed two times by PBS. Finally, the conidia were suspended in fresh cold PBS, stored at 4 °C, and used within 1 month.

### Conidia infection of hPEOs

Because the gel can act as a diffusion barrier for *T. rubrum*, to improve their access to the organoids, the hPEOs (at 7–9 days of culture), were firstly released from BME 2 or Matrigel through incubating the 3D drops on ice for 30–60 min. Then, the isolated hPEOs were re-suspended in fresh BME 2 or Matrigel that contained conidia at a density of 2 × 10^5^ per 30 µL gel. After the gel solidification, they were covered by organoids growth medium and incubated at 37 °C.

### mRNA sequencing and analysis

The hPEOs at 24 h after infection or in controls, without infection, were released from BME 2 or Matrigel by incubating the 3D drops on ice for 30–60 min and washed by PBS as much as possible to remove the adhering hyphae. The total RNA of the infected hPEOs and non-infected hPEOs were extracted using Trizol according to the manufacturer’s instructions. RNA-Seq libraries were generated using NEBNext Ultra RNA Library Prep Kit for Illumina (NEB, USA). Sequencing was performed by Annoroad (China). RNA-Seq was sequenced on Noves6000 platform. The reads were mapped to the human reference genome (hg4) using HISAT2. The low-quality parts of raw reads were filtered. Clean Data was aligned to the reference genome using HISAT2 v2.1.0. Reads Count for each gene in each sample was counted by HTSeq v0.6.0, and FPKM (Fragments Per Kilobase Millon Mapped Reads) was then calculated to estimate the expression level of genes in each sample. Differentially expressed genes (DEGs) were analyzed by DESeq2 using counts. Genes with *P* < 0.05 and FC > 1.5 are identified as DEGs. Original data were uploaded to the Gene Expression Omnibus database (accession number GSE134403).

### Transmission electron microscopy

The *T.* rubrum-infected or non-infected hPEOs were fixed with 2.5% glutaraldehyde at 4 °C overnight. Then the cells were post-fixed by incubation for 2 h with 1% osmium tetroxide/0.1 sodium cacodylate and dehydrated in a graded series of acetone solutions. The cells were embedded in Polybed 812 epoxy resin (Polysciences, Inc, Warrington, PA). Ultrathin sections were cut and collected on 50 mesh copper grids, stained with 4% aqueous uranyl for 15 min, and then with Reynolds’ lead citrate for 7 min. Stained sections were examined with a JEM-1400 Plus electron microscope (JEOL).

### Western blots

Protein concentrations of cell lysates lysed in RIPA (Beyotime Biotechnology) or concentrated conditional medium derived from *T. rubrum* group and control group were measured using a Pierce BCA Protein Assay Kit (Thermo Fisher Scientific). Proteins were subjected to electrophoresis on 15% Bis-Tris gels and transferred to polyvinylidene difluoride (PVDF) membranes (Millipore), which were incubated with the primary antibodies, followed by a secondary antibody. The antibodies are listed in Supplementary Table 1.

### Statistical analysis

The data were shown as mean ± SD and *P* value were calculated by two-tailed Student’s *t* test by the GraphPad Prism 8.0 software. n.s., not significant (*p* > 0.05), *p < 0.05, **p < 0.01, ***p < 0.001, ****p < 0.0001. Each quantitative experiment was repeated at least three times.

## Supplementary information

Supplementary Figure Legends

Supplementary figure 1

Supplementary figure 2

Supplementary figure 3

Supplementary figure 4

Supplementary figure 5

Supplementary figure 6

Supplementary Table 1

Supplementary Table 2
