# Supplementary material for: Human primary epidermal organoids enable modeling of dermatophyte infections
Source: Cell Death Dis. 2021 Jan 4;12(1):35. doi: 10.1038/s41419-020-03330-y (PMC7790817; doi:10.1038/s41419-020-03330-y)
Supplement: Supplementary file 1 — Supplementary Figure Legends [file 41419_2020_3330_MOESM1_ESM.docx]

**Supplementary Fig. 1** Evaluation of the viability of cells derived from the novel method and the traditional method by Fixable Viability Stain 510.

**Supplementary Fig. 2** Representative images of hPEOs after removal of individual factors from the NaNBEFNoRWAFs pool. Scale bar: 100µm.

**Supplementary Fig. 3** Representative images of hPEOs after removal of individual factors from the pool of NaBEFWAFs. Scale bar: 100µm.

**Supplementary Fig. 4** Representative images of hPEOs after removal of individual factors from the pool of NaBEWAFs. Scale bar: 100µm.

**Supplementary Fig. 5** Skin special markers for sections of primary epidermis tissue and hPEOs. **A-D** Immunostaining of basal cell markers, CK5, ITGB4 (**A**), suprabasal marker, CK10 (**B**), basement membrane components, Lam, Col Ⅳ, Col Ⅶ (**C**), non-epidermal cell markers, Gp100, CD1a, CD31, Vimentin (**D**) for sections of primary epidermis tissue and hPEOs.Gp100, melanoma gp100. Dotted line marks the epidermis–dermis junction. Scale bar: 50µm.

**Supplementary Fig. 6** The cellular responses of hPEOs to *T. rubrum* infections. **A** Heatmap showing the expression of epidermal differentiation and cornification-associated genes in Control and *T. rubrum* group. **B** Analysis of protein level of IL-1RN by western blot assays in Control and *T. rubrum* group post 24h infection. **C** Immunohistochemical staining for IL-36RN in hPEOs of Control group and *T. rubrum* group. **D, E** qRT–PCR analysis of the expression of cell surface TLRs (**D**) and human beta defensins (**E**) altered in the organoid in response to *T rubrum* infections. n.s., not significant (p>0.05), *p<0.05, **p<0.01, ***p<0.001, ****p<0.0001. Scale bar: 50µm.
