# Supplementary figures and images for: Human primary epidermal organoids enable modeling of dermatophyte infections
Source: Cell Death Dis. 2021 Jan 4;12(1):35. doi: 10.1038/s41419-020-03330-y (PMC7790817; doi:10.1038/s41419-020-03330-y)

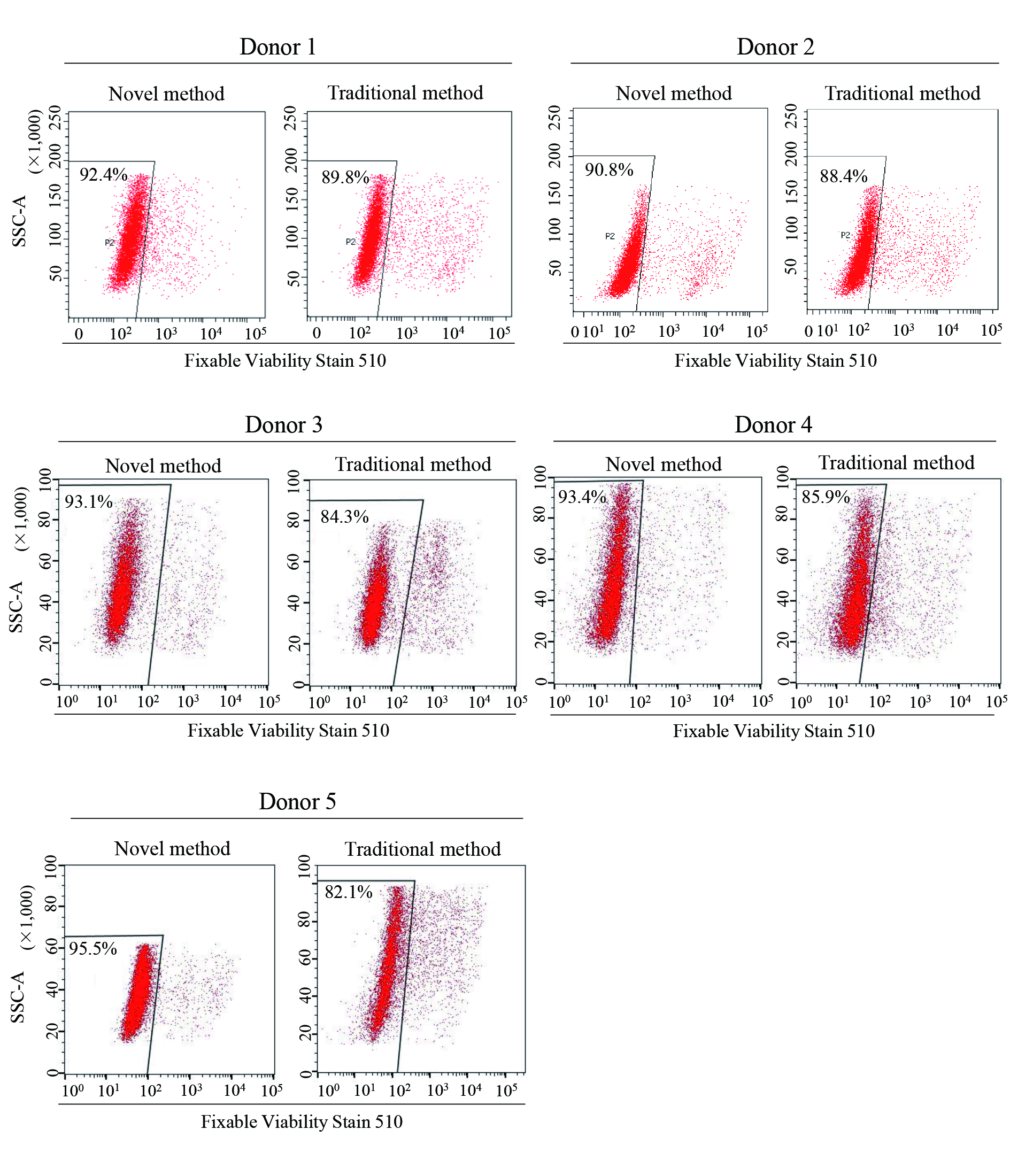

Supplement: Supplementary file 2 — Supplementary figure 1 [file 41419_2020_3330_MOESM2_ESM.tif]

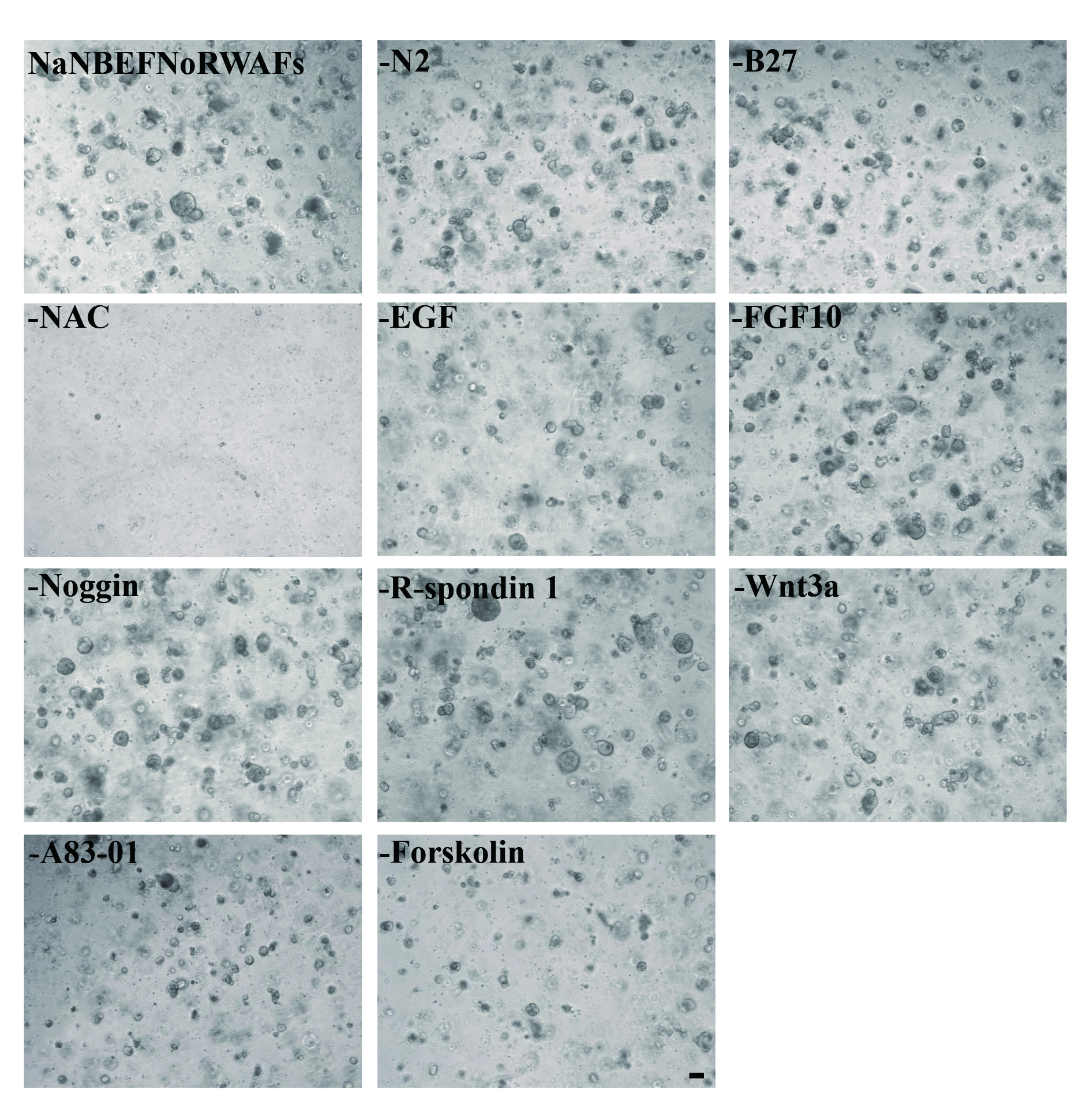

Supplement: Supplementary file 3 — Supplementary figure 2 [file 41419_2020_3330_MOESM3_ESM.tif]

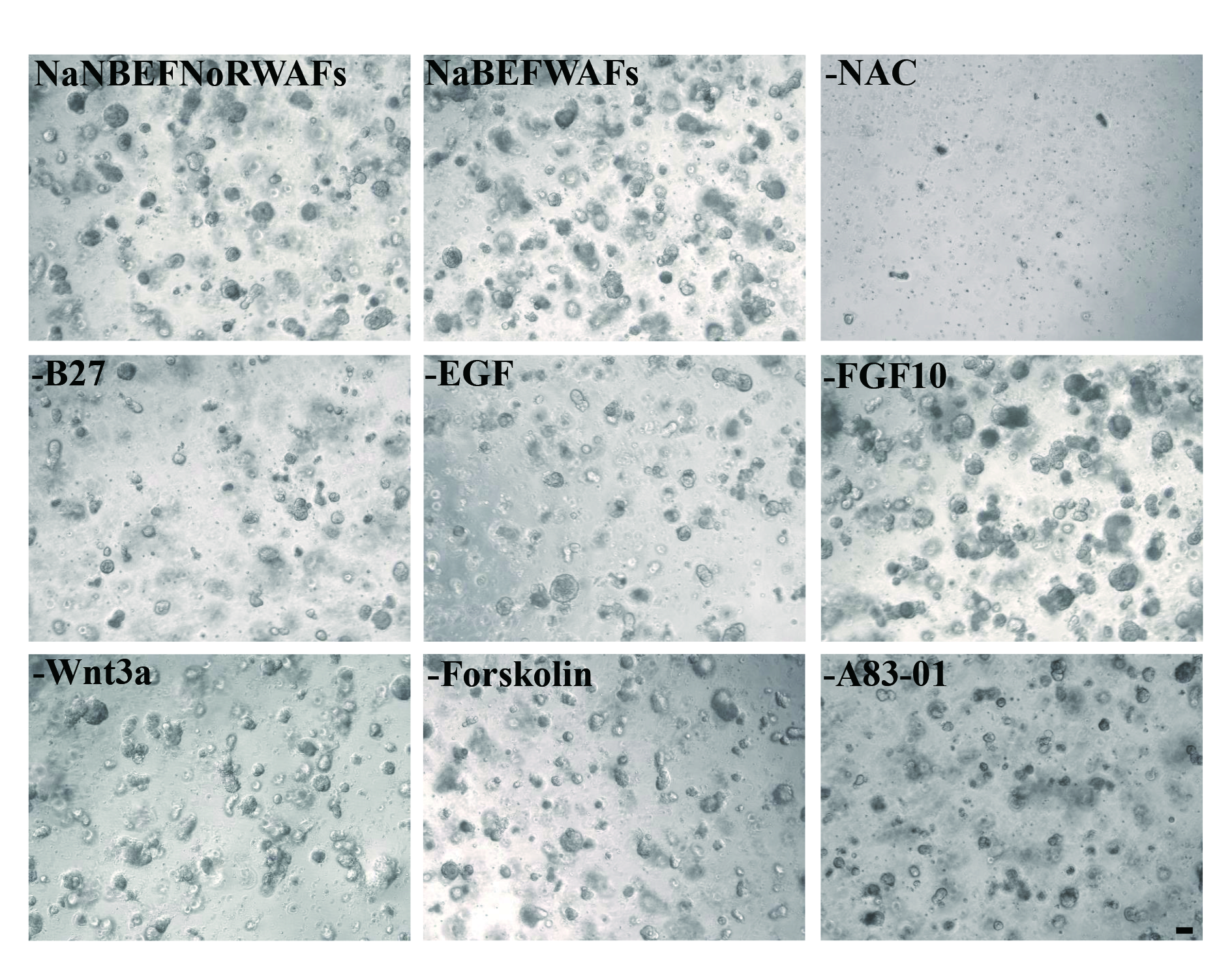

Supplement: Supplementary file 4 — Supplementary figure 3 [file 41419_2020_3330_MOESM4_ESM.tif]

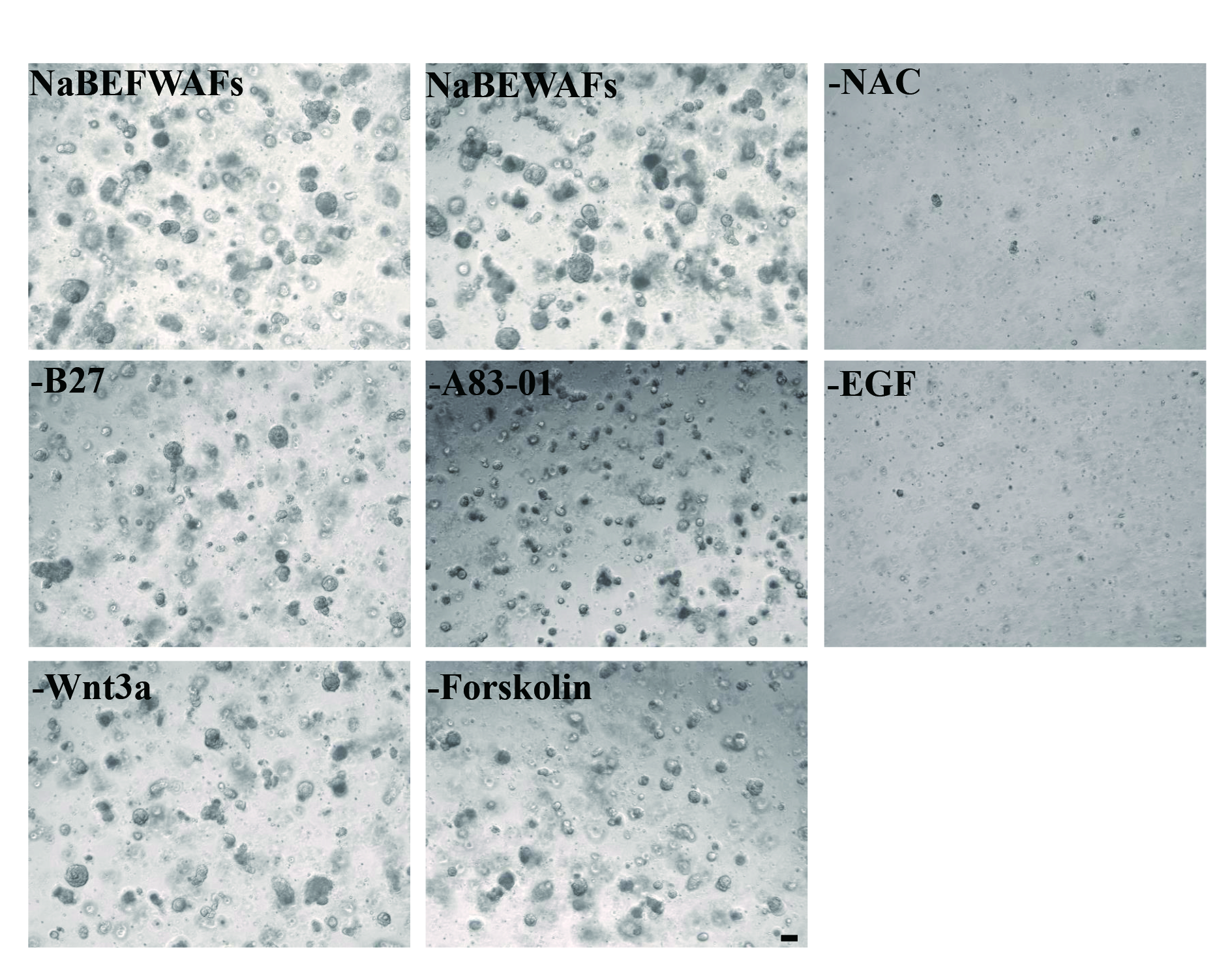

Supplement: Supplementary file 5 — Supplementary figure 4 [file 41419_2020_3330_MOESM5_ESM.tif]

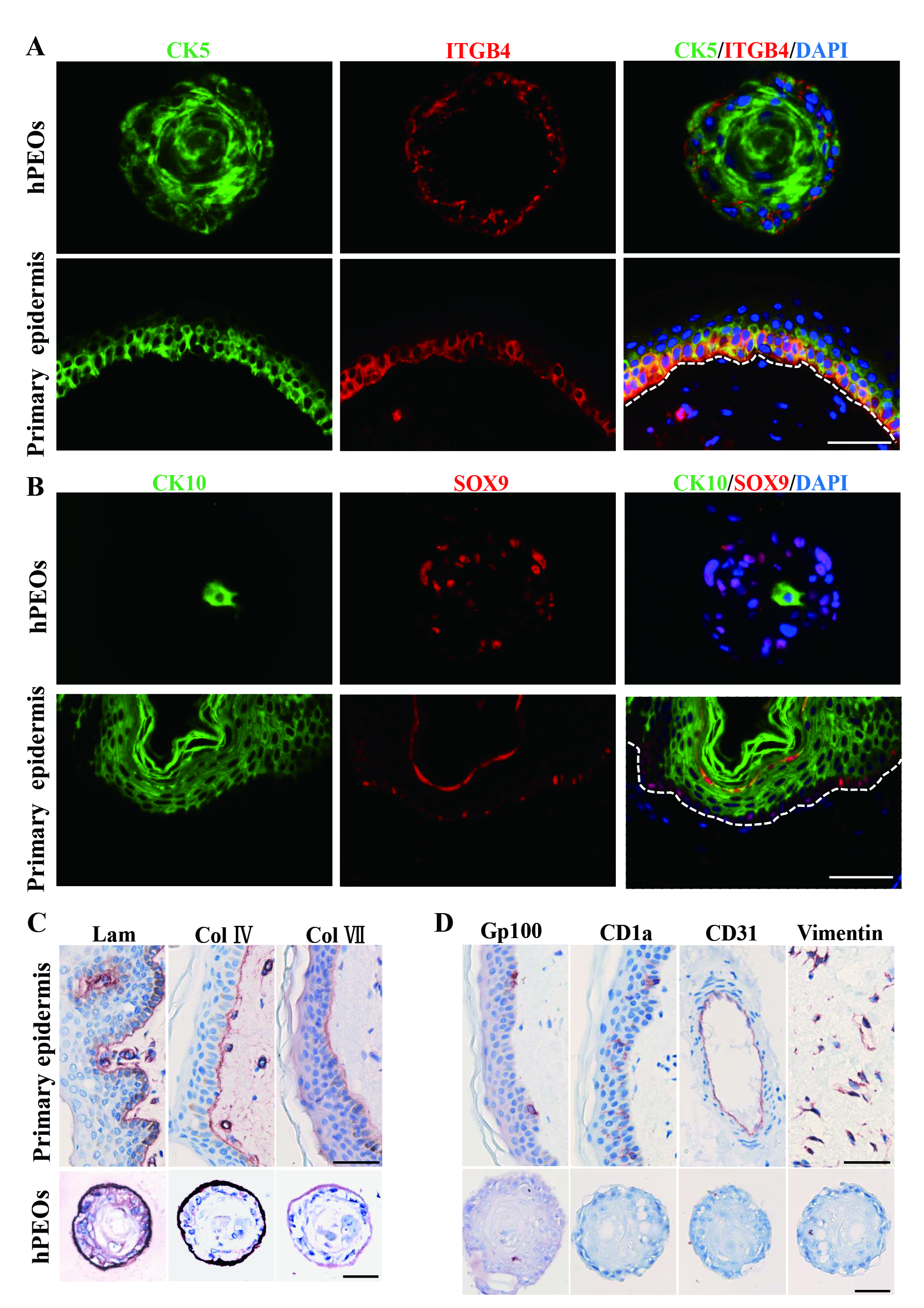

Supplement: Supplementary file 6 — Supplementary figure 5 [file 41419_2020_3330_MOESM6_ESM.tif]

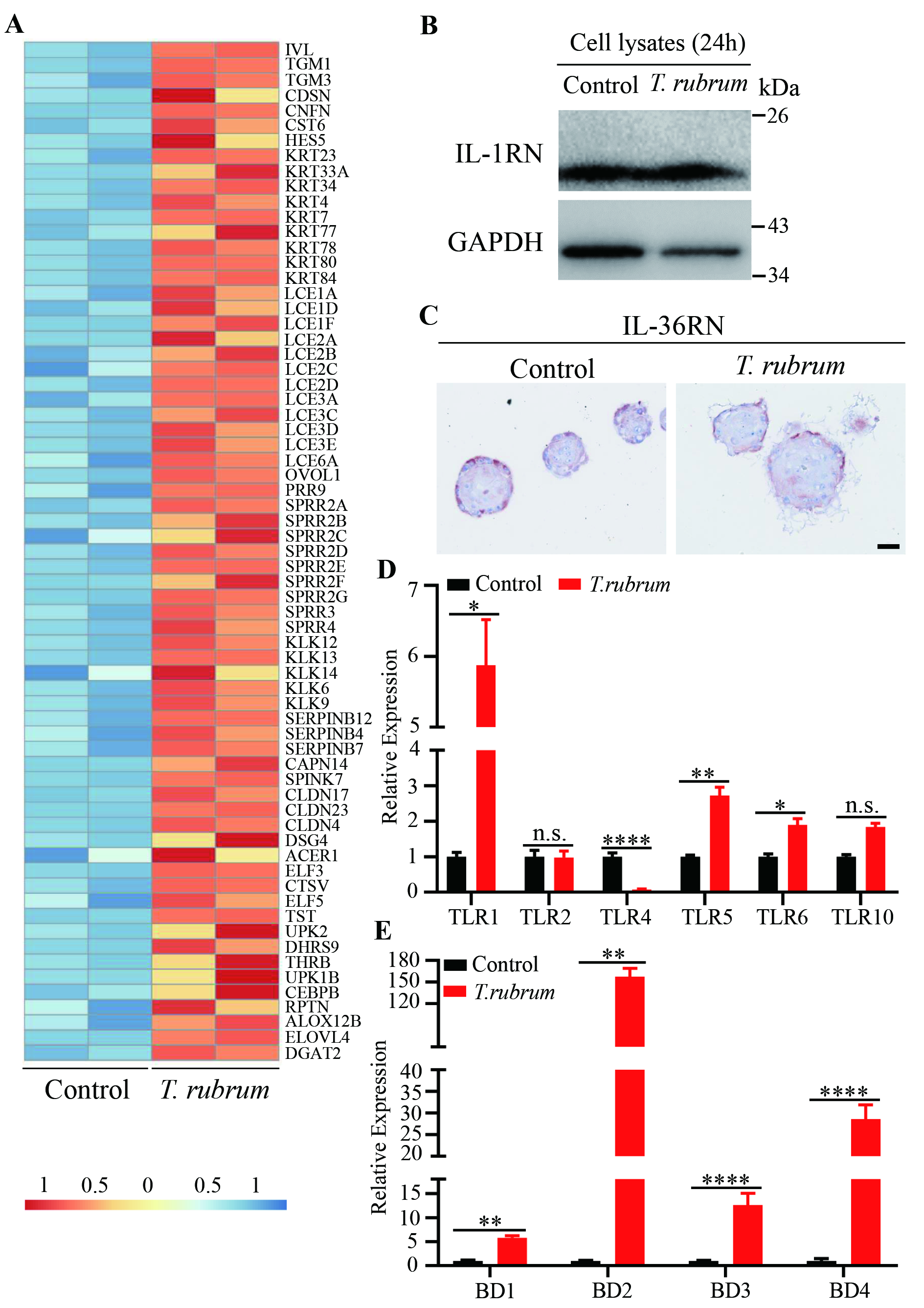

Supplement: Supplementary file 7 — Supplementary figure 6 [file 41419_2020_3330_MOESM7_ESM.tif]
