# Supplementary material for: Human primary epidermal organoids enable modeling of dermatophyte infections
Source: Cell Death Dis. 2021 Jan 4;12(1):35. doi: 10.1038/s41419-020-03330-y (PMC7790817; doi:10.1038/s41419-020-03330-y)
Supplement: Supplementary file 8 — Supplementary Table 1 [file 41419_2020_3330_MOESM8_ESM.doc]

**Table S1. Antibody list**

| Primary antibody | | | | |
| --- | --- | --- | --- | --- |
| Antibody | Company | Product number | Ig Species | Dilution |
| CK1 | Abcam | ab93652 | Rabbit IgG | 100 |
| CK1 | Santa Cruz | sc-376224 | Mouse IgG2a | 100 |
| CK5 | Abcam | ab53121 | Rabbit IgG | 800 |
| CK10 | Abcam | ab76318 | Rabbit IgG | 1000 |
| CK14 | Abcam | ab9220 | Mouse IgG1 | 200 |
| CD1a | Abcam | ab201337 | Mouse IgG1 | 200 |
| CD31 | Abcam | ab9498 | Mouse IgG1 | 50 |
| Col IV | Abcam | ab6586 | Rabbit IgG | 500 |
| Col VII | Abcam | ab6312 | Mouse IgG1 | 200 |
| Claudin1 | Abcam | ab15098 | Rabbit IgG | 200 |
| E-cadherin | Abcam | ab76055 | Mouse IgG1 | 200 |
| GP100 | Abcam | ab190731 | Mouse IgG1 | 200 |
| Involucrin | Abcam | ab53112 | Rabbit IgG | 50 |
| Filaggrin | Santa Cruz | sc-66192 | Mouse IgG1 | 100 |
| Integrin β4 | Abcam | ab29042 | Mouse IgG1 | 100 |
| Integrin α6 | Abcam | ab20142 | Mouse IgG2b | 100 |
| Ki67 | eBioscience | 13-5698 | Rat IgG 2a | 200 |
| Laminin | Abcam | ab11575 | Rabbit IgG | 50 |
| Vimentin | Abcam | ab8978 | Mouse IgG1 | 200 |
| P63 | Abcam | ab7754 | Mouse IgG2a | 50 |
| B-catenin | Abcam | ab32572 | Rabbit IgG | 500 |
| DSC2 | Proteintech | 13876-1-AP | Rabbit IgG | 200 |
| IL-1RN | Abcam | ab124962 | Rabbit IgG | 100 |
| IL-36RN | Abcam | ab224489 | Rabbit IgG | 500 |
| Conjugated antibody | | | | |
| Antibody | Company | Code number | Ig species |  |
| PE-Rat Anti-Human CD49f | BD Biosciences | 555736 | Rat IgG2a |  |
| Secondary antibody | | | | |
| Secondary antibody | | Company | Code number | Dilution |
| Alexa Fluor® 568 Goat Anti-Mouse IgG1 (γ1) | | Invitrogen | A21124 | 400 |
| Alexa Fluor® 488 Goat Anti-Mouse IgG2a (γ2a) | | Invitrogen | A21131 | 400 |
| Alexa Fluor® 647 Goat Anti-Mouse IgG2b (γ2b) | | Invitrogen | A21242 | 400 |
| Alexa Fluor® 647 Donkey Anti-Mouse IgG (H+L) | | Invitrogen | A31571 | 400 |
| Alexa Fluor® 568 Donkey Anti-Mouse IgG (H+L) | | Invitrogen | A10037 | 400 |
| Alexa Fluor® 488 Donkey Anti-Mouse IgG (H+L) | | Invitrogen | A21202 | 400 |
| Alexa Fluor® 568 Donkey Anti-Goat IgG (H+L) | | Invitrogen | A11057 | 400 |
| Alexa Fluor® 488 Donkey Anti-Rabbit IgG (H+L) | | Invitrogen | A21206 | 400 |
| Alexa Fluor® 568 Goat Anti-Mouse IgG1 (γ1) | | Invitrogen | A21124 | 400 |
| Alexa Fluor 647 Goat Anti-Rat IgG (H+L) | | Invitrogen | A21247 | 400 |
