# Supplementary material for: Human primary epidermal organoids enable modeling of dermatophyte infections
Source: Cell Death Dis. 2021 Jan 4;12(1):35. doi: 10.1038/s41419-020-03330-y (PMC7790817; doi:10.1038/s41419-020-03330-y)
Supplement: Supplementary file 9 — Supplementary Table 2 [file 41419_2020_3330_MOESM9_ESM.doc]

**Table S2.** Primer list

| Gene | Forward | Reward |
| --- | --- | --- |
| GAPDH | GAGTCAACGGATTTGGTCGT | TTGATTTTGGAGGGATCTCG |
| CK5 | ATCTCTGAGATGAACCGGATGATC | CAGATTGGCGCACTGTTTCTT |
| CK14 | GGCCTGCTGAGATCAAAGACTAC | CACTGTGGCTGTGAGAATCTTGTT |
| Integrin β4 | CTGTACCCGTATTGCGACT | AGGCCATAGCAGACCTCGTA |
| Integrin α6 | GCTGGTTATAATCCTTCAATATCAATTGT | TTGGGCTCAGAACCTTGGTTT |
| P63 | GGACCAGCAGATTCAGAACGG | AGGACACGTCGAAACTGTGC |
| SOX9 | AGCGAACGCACATCAAGAC | GCTGTAGTGTGGGAGGTTGAA |
| Ki67 | ACGCCTGGTTACTATCAAAAGG | CAGACCCATTTACTTGTGTTGGA |
| IL-36G | AGGAAGGGCCGTCTATCAATC | CACTGTCACTTCGTGGAACTG |
| IL-1RN | CATTGAGCCTCATGCTCTGTT | CGCTGTCTGAGCGGATGAA |
| IL-IF10 | AGAGGGGCCTTCCCTACAG | CAGCAGCCTCAAGCCTGAA |
| IL-36B | ATGAACCCACAACGGGAGG | TAATGCTGCGGCTAAGAGGAG |
| IL-36RN | ACTCGGCATTGAAGGTGCTTT | GGGACCACGCTGATCTCTT |
| TLR1 | TGAACCTCAAGCACTTGGACC | CCCATAAGTCTCTCCTAAGACCA |
| TLR2 | ATCCTCCAATCAGGCTTCTCT | GGACAGGTCAAGGCTTTTTACA |
| TLR4 | AGTTGATCTACCAAGCCTTGAGT | GCTGGTTGTCCCAAAATCACTTT |
| TLR5 | TCCCTGAACTCACGAGTCTTT | GGTTGTCAAGTCCGTAAAATGC |
| TLR6 | TGAATGCAAAAACCCTTCACCT | CCAAGTCGTTTCTATGTGGTTGA |
| TLR10 | GATTTACTCTGGGACGACCTTTT | GTCAAGATAAGCCTTACCACCAA |
| hBD-1 | ATGAGAACTTCCTACCTTCTGCT | TCTGTAACAGGTGCCTTGAATTT |
| hBD-2 | GGTGGTATAGGCGATCCTGTT | AGGGCAAAAGACTGGATGACA |
| hBD-3 | TCCAGGTCATGGAGGAATCAT | CGAGCACTTGCCGATCTGT |
| hBD-4 | AGACTTGTGCTGCTATTAGCCG | GGGCAGTCCCATAACCACATA |
